# Taxonomy and phenotypic relationships of the *Anastrepha fraterculus* complex in the Mesoamerican and Pacific Neotropical dominions (Diptera, Tephritidae)

**DOI:** 10.3897/zookeys.540.6027

**Published:** 2015-11-26

**Authors:** Vicente Hernández-Ortiz, Nelson A. Canal, Juan O. Tigrero Salas, Freddy M. Ruíz-Hurtado, José F. Dzul-Cauich

**Affiliations:** 1 Instituto de Ecología A.C., Red de Interacciones Multitróficas. Carretera Antigua a Coatepec 351, El Haya CP 91070. Xalapa, Veracruz, México; 2 Universidad del Tolima, Facultad de Ingeniería Agronómica. Barrio Santa Helena parte alta, CP 730006299, Ibagué, Tolima, Colombia; 3 Universidad de las Fuerzas Armadas (ESPE), Departamento Ciencias de la Vida, Ingeniería Agropecuaria. Campus Politécnico Av. Gral. Rumiñahui s/n, PO Box 171-5- 231B, Sangolquí, Ecuador

**Keywords:** Cryptic species complex, linear morphometrics, geometric morphometrics, distribution

## Abstract

Previous morphometric studies based on linear measurements of female structures of the aculeus, mesonotum, and wing revealed the existence of seven morphotypes within the *Anastrepha fraterculus* cryptic species complex along the Neotropical Region. The current research followed linear and geometric morphometric approaches in 40 population samples of the nominal species *Anastrepha fraterculus* (Wiedemann) spread throughout the Meso-American and Pacific Neotropical dominions (including Mexico, Central America, Venezuela, Colombia, Ecuador, and Peru). The goals were to explore the phenotypic relationships of the morphotypes in these biogeographical areas; evaluate the reliability of procedures used for delimitation of morphotypes; and describe their current distribution. Findings determined that morphotypes previously recognized via the linear morphometrics were also supported by geometric morphometrics of the wing shape. In addition, we found an eighth morphotype inhabiting the highlands of Ecuador and Peru. Morphotypes are related into three natural phenotypic groups nominated as Mesoamerican-Caribbean lineage, Andean lineage, and Brazilian lineage. The hypothesis that lineages are not directly related to each other is discussed, supported by their large morphological divergence and endemicity in these three well-defined biogeographic areas. In addition, this hypothesis of the non-monophyly of the *Anastrepha fraterculus* complex is also supported by evidence from other authors based on molecular studies and the strong reproductive isolation between morphs from different lineages.

## Introduction

Phylogenetic relationships stated that infrageneric classification based on morphology of the genus *Anastrepha* Schiner recognizes nearly 21 species groups (Norrbom et al. 1999, 2012). One of them is the *“fraterculus* species group” consisting of 34 species, with some widely distributed in the Neotropics (e.g., *Anastrepha ludens* (Loew), *Anastrepha obliqua* (Macquart), *Anastrepha suspensa* (Loew), and *Anastrepha fraterculus*). One of these nominal species, the “South American fruit fly” *Anastrepha fraterculus* (Wiedemann) occurs from south of the USA (Texas) through Mexico, Central America to Argentina and represents a cryptic species complex (hereafter denoted as the *Af* complex).

First evidence of the *Af* cryptic species complex appeared in the comprehensive taxonomic revision of the genus *Anastrepha* made by Stone (1942). Since then, other findings from distinct populations along its distributional range were reported supporting this hypothesis, such as differences of karyotypes (Mendes 1958, Bush 1962, Solferini and Morgante 1987, Selivon et al. 2005b); isozyme divergence (Morgante et al. 1980, Steck 1991); DNA sequences (Steck and Sheppard 1993, Smith-Caldas et al. 2001); or studies with multiple approaches including karyotype, isozymes, and morphology (Selivon and Perondini 1998, Selivon et al. 2004, 2005a). Moreover, differences in host range and pest status (Baker 1945, Aluja et al. 2003, Hernández-Ortiz and Morales-Valles 2004, Zucchi 2007); reproductive isolation and sexual incompatibilities (Selivon et al. 1999, 2005a, Vera et al. 2006, Cáceres et al. 2009, Rull et al. 2013, Devescovi et al. 2014); or data on pheromone composition and cuticular hydrocarbon profiles (Břízová et al. 2013, Vaníčková et al. 2015).

Morphometric analyses have been a useful technique in detecting morphological differences among organisms to distinguish closely related species of fruit flies (Adsavakulchai et al. 1999, Khamis et al. 2012, Schutze et al. 2012). Based on adult morphology of the *Af* complex, Hernández-Ortiz et al. (2004) developed a morphometric technique using linear measurements of the aculeus, wing, and mesonotum for the full recognition of the Mexican morphotype, separating it from other South American samples from Colombia, Brazil and Argentina. Further linear morphometric studies applied to 32 populations from Mexico, Central America, and South America (including Venezuela, Colombia, Ecuador, Peru, Brazil and Argentina) confirmed previous findings, and added the fact that seven morphotypes could be distinguished within the *Af* complex throughout the Neotropical region (Hernández-Ortiz et al. 2012).

Despite all evidence gathered by different sources, it is still difficult to set out the taxonomic status of the morphotypes mainly due to two reasons. The first one is that other methodological approaches, such as DNA sequences or sexual compatibility have shown large interpopulation divergences, without allowing full identification of interspecific boundaries; and the second one is that information about the overall distribution of the cryptic species still remains uncertain. This is especially true for morphotypes occurring in the North and Central Andes, and for the Brazilian morphotypes.

According to Daly (1985), multivariate methods of morphometric analysis (e.g., DFA, PCA) can be widely applied in biology. However, two general kinds of problems may be encountered in canonical variate analysis of morphometric data: a) linear dependence when two or more variables are highly correlated; and b) heteroscedasticity of the covariance matrices (inequality of dispersion matrices). In this sense, ratios have been used for scaling morphometric variables to remove variation in general body size; to express shape by finding the proportion of one dimension of a structure to another; and to express growth in the size of some structure from one instar to the next. Additionally, because linear distance measurements usually are highly correlated with size, much effort was spent in developing methods for size correction, so that size-free shape variables could be extracted and patterns of shape variation elucidated (Bookstein et al. 1985, Sundberg 1989). The most widespread approach of the geometric morphometrics, is to represent each specimen by the relative positions of morphological landmarks, that can be located precisely and establish a one-to-one correspondence among all specimens included in the analysis (Klingenberg 2010). Shape is defined as all the geometric information about a configuration of landmarks and it is extracted by a procedure called Procrustes superimposition, which removes variation in size, position and orientation from the data on landmark coordinates, and which is at the core of geometric morphometrics (Goodall 1991, Dryden and Mardia 1998, Zelditch et al. 2012).

Another crucial issue for the resolution of this cryptic species complex is understanding the distributional patterns of their morphotypes. Morrone (2014) recently revised the biogeographic regionalization of the Neotropical region. The Mesoamerican dominion comprises lowlands of central and southern Mexico, and most of Central America (Guatemala, Belize, Honduras, El Salvador and northern Nicaragua). The Pacific dominion encompasses southern Central America (southeastern Nicaragua to Panama) and northwestern South America (including western Colombia, Ecuador, Peru, northwestern Venezuela, Trinidad and Tobago, and the Galapagos Islands). Contiguous to these dominions, the Mexican Transition Zone (MTZ) occupies an area where the Neotropical and Nearctic regions overlap, corresponding basically to the mountainous areas of central and southern Mexico and northern Central America; and the South American Transition Zone (SATZ) represented by highlands of the Andes between western Venezuela and northern Chile, and central western Argentina (*sensu* Morrone 2006). In this sense, correlating the occurrence of the different morphotypes to biogeography will add valuable information to delimit the distribution of the species involved.

Given this scenario, systematic studies that identify the incidence areas of the different *Af* morphotypes throughout the Neotropical region are needed. Increasing the number of samples from Colombia, Ecuador and Peru will confirm previous evidence that suggests that biogeographical and ecological factors in these countries, contribute to the understanding of the distributional patterns of the morphotypes. As such, the goals of this study were to explore phenotypic relationships among different morphs of the *Af* complex in the Mesoamerican and Pacific biogeographical dominions; to make comparisons of the usefulness of the linear morphometrics and geometric morphometry of the wing shape for delimitation of the morphotypes; and to describe their distributional patterns throughout the biogeographical provinces currently recognized.

## Methods

### Biological material

We used samples from forty populations obtained from different sources. Most of them were collected from nature directly on their hosts and afterwards reared to adult specimens in the laboratory. Others were collected in McPhail traps baited with hydrolyzed protein, and in few cases, we analyzed samples from laboratory strains established for long time at the Seibersdorf facilities of the FAO/IAEA Agriculture and Biotechnology Laboratories (Austria). Specific data of collection, country, location, and specimens examined are listed in Table 1.

**Table 1.** List of samples examined of the *Anastrepha fraterculus* along the Neotropics, showing data of location, georeferentiation, and source of sample

Sample-Key	Country	Locality	Altitude (m)	Coordinates	Source	N Linear	N Geometrics
MEX-Jica	Mexico	La Jicayana	400	19°21'44"N, 96°39'23"W	McPhail trap	10	10
MEX-Teoc	Mexico	Tejería	980	19°23'14"N, 96°36'59"W	*Psidium guineense*	10	10
MEX-Apaz	Mexico	Apazapan	250	19°17'00"N, 96°39'23"W	McPhail trap	10	10
MEX-Coat	Mexico	Coatepec	1200	19°27'25"N, 96°57'29"W	*Syzygium jambos*	10	10
MEX-Tuxt	Mexico	Los Tuxtlas	160	18°35'06"N, 95°04'12"W	*Psidium guajava*	10	10
MEX-Chis	Mexico	San Vicente	1400	16°11'50"N, 92°02'57"W	*Psidium guajava*	10	10
MEX-Tap	Mexico	Tapachula	150	ND	Seibersdorf Lab-strain	6	6
MEX-QRoo	Mexico	Chunhuhub	30	19°37'39"N, 88°38'56"W	McPhail trap	10	10
GUA-City	Guatemala	Guatemala City	1500	14°36'51"N, 90°32'22"W	*Psidium guajava*	15	15
PAN-LCam	Panama	La Campana	61	08°44'16"N, 79°51'29"W	*Psidium guajava*	15	15
PAN-BCol	Panama	Barro Colorado Is.	125	09°09'08"N, 79°50'47"W	*Eugenia uniflora*	17	17
VEN-Corr	Venezuela	Corrales	40	10°44'35"N, 71°21'10"W	McPhail trap	15	15
VEN-LMit	Venezuela	Loma Mitimbís	1570	09°16'57"N, 70°14'59"W	*Rubus glaucus*	15	15
VEN-DDiaz	Venezuela	Diego Díaz	1640	ND	*Eriobotrya japonica*	15	15
VEN-SDom	Venezuela	Santo Domingo	2500	08°57'37” N, 71°02'54"W	*Coffea arabica*	15	15
VEN-Tig	Venezuela	Tiguanín	1900	ND	*Psidium caudatum*	15	15
COL-Cund	Colombia	La Mesa	1350	04°38'09"N, 74°27'21"W	McPhail trap	10	10
COL-Tol	Colombia	Vereda Gamboa	1600	04°26'11"N, 75°11'29"W	Seibersdorf Lab-strain	15	15
COL-Bar	Colombia	Barbosa	1880	05°55'57"N, 73°37'16"W	*Psidium guajava*	13	16
COL-Cach	Colombia	Tocarema alto	1850	04°45'01"N, 74°23'01"W	*Coffea arabica*	20	20
COL-Duit	Colombia	Duitama	2569	05°49'29"N, 73°04'29"W	*Acca sellowiana*	20	20
COL-Rold	Colombia	La Aguada	1764	04°23'05"N, 76°13'20"W	*Coffea arabica*	20	20
COL-Lun	Colombia	El Guabo	1704	01°36'53"N, 77°07'53"W	*Coffea arabica*	20	20
COL-Pen	Colombia	Pensilvania	2091	05°22'03"N, 75°09'29"W	*Acca sellowiana*	20	21
COL-Sev	Colombia	Sevilla	1556	04°17'19"N, 75°54'23"W	*Coffea arabica*	20	20
COL-Sibu	Colombia	Fatima	2136	01°12'05"N, 76°54'48"W	*Psidium acutangulum*	20	20
COL-Ibag	Colombia	Ibagué	1433	04°24'53"N, 75°18'50"W	*Lab colony-U Tolima*	20	20
ECU-Agro	Ecuador	Km39 via la Costa	7	01°57'15"S, 79°55'17"W	McPhail trap	17	20
ECU-Guay	Ecuador	Guayaquil	80	02°12'13"S, 79°53'50"W	*Psidium guajava*	14	15
ECU-Bab	Ecuador	Recinto Tauín	91	01°45'29"S, 79°26'50"W	McPhail trap	20	20
ECU-Chac	Ecuador	Chacras	370	03°26'51"S, 79°49'53"W	McPhail trap	20	20
ECU-Chot	Ecuador	Ambuquí	1550	00°26'50"N, 78°00'18"W	McPhail trap	20	20
ECU-Per	Ecuador	Perucho	1861	00°06'48"N, 78°25'33"W	McPhail trap	20	20
ECU-Pich	Ecuador	Guayllabamba	2176	00°03'47"S, 78°20'56"W	McPhail trap	20	20
ECU-Pat	Ecuador	Patate	2034	01°19'04"S, 78°30'44"W	McPhail trap	20	20
PER-Piu	Peru	Piura	35	05°12'00"S, 80°37'00"W	Seibersdorf Lab-strain	15	15
PER-LMol	Peru	La Molina	300	12°05'21"S, 76°55'41"W	Seibersdorf Lab-strain	15	15
PER-Chon	Peru	Chongona	1502	12°45'49"S, 72°36'15"W	McPhail trap	7	10
PER-Echa	Peru	Puente Echarate	941	12°46'10"S, 72°34'37"W	McPhail trap	11	14
PER-VSag	Peru	Valle Sagrado	2859	13°19'00"S, 72°05'21"W	McPhail trap	17	17

Permanent mounting slides were made prior to observations. Female aculeus was cleaned in a boiling solution, consisting of 10% sodium hydroxide, for approximately 15–20 min; in addition the right wing of each specimen was cut from its base. After that, structures were washed with distilled water and further dehydrated by gradual alcohol series (50, 70, 100% by holding them for 20 min at each step), placed in xylene 2–3 min, and immediately mounted with Canada balsam. Digital images of the mesonotum and wing were made with a digital camera (Olympus C5050) adapted to stereomicroscope (Olympus SZX7); and images of the aculeus were performed using an optical microscope (Olympus BX41) with objective 40X. Permanent slides and pinned voucher specimens of the studied samples were deposited at the Entomological Collections of the Instituto de Ecología AC (Xalapa, Mexico), Universidad del Tolima (Ibagué, Colombia), and the Universidad de las Fuerzas Armadas – ESPE (Quito, Ecuador).

### Linear Morphometrics

**Figures 1–4.**
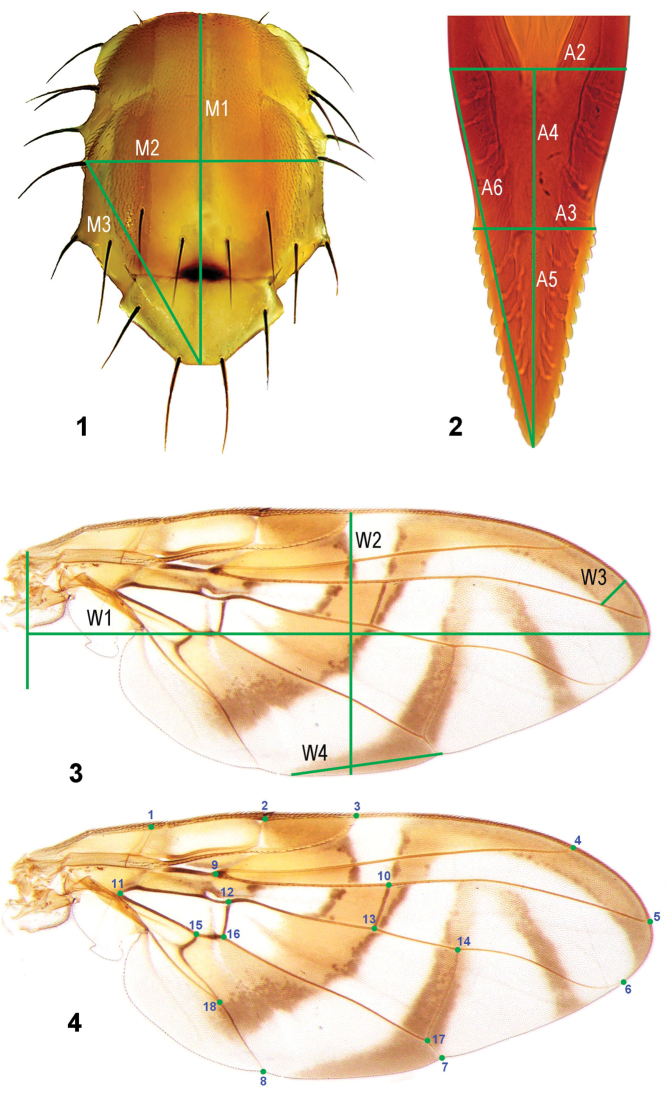
Morphological structures and variables used for morphometric assessment of the *Anastrepha fraterculus* complex: **1** Thorax in dorsal view **2** Aculeus tip in ventral view **3** Measurements for the linear morphometrics of wing **4** Landmarks used for the geometric analysis of the wing shape.

In total, 612 female specimens were examined, considering 27 morphometric traits of structures such as mesonotum, aculeus, and wing. Variables as linear distances between two points, as ratios of two variables, and qualitative features of wing pattern were assessed following methods described by Hernández-Ortiz et al. (2004, 2012):

*Mesonotum* (Figure 1). M1) mesonotal length; M2) mesonotal width at level of postsutural supra-alar seta; M3) length from the apex of scutellum to the left postsutural supra-alar seta.

*Aculeus* (Figure 2). A1) total aculeus length; A2) basal width of the aculeus tip; A3) width at beginning of serrated section; A4) basal tip length of non-serrated section; A5) apical tip length of serrated section; A6) length from basal left side to aculeus apex; A7) mean number of lateral teeth; A8) aculeus tip length (A4+A5); A9) ratio of the length of nonserrated section/length of serrated section (A4/A5); A10) ratio of aculeus tip length/aculeus length (A8/A1); A11) ratio of length of non-serrated section/aculeus tip length (A4/A8).

*Wing* (Figure 3). W1) wing length; W2) wing width at R_1_ apex; W3) width of apical section of S-band (from juncture of S-band and vein R_4+5_ perpendicular to Costal vein); W4) distance from proximal end of proximal arm of V-band on posterior wing margin to apex of vein Cu1; W5) S- and V-band connection between R_2+3_ and R_4+5_ (1 = present; 2 = absent); W6) V-band anterior connection of proximal and distal arms between R_4+5_ and M (1 = present; 2 = absent); W7) ratio of wing width/wing length (W2/W1).

Additional variables of ratios between two measurements were assessed as follows: X1) aculeus length/mesonotum length (A1/M1); X2) aculeus length/wing length (A1/W1); X3) mesonotum length/wing length (M1/W1); X4) mesonotum length/mesonotum width (M1/M2); X5) width at beginning of serrated section/length of serrated section (A3/A5); X6) mesonotum width/wing length (M2/W1).

### Geometric morphometrics

Eighteen homologous landmark coordinates were digitized on the wings. A total as 626 females belonging to 40 populations distributed from Mexico through Central America, Venezuela, Colombia, Ecuador and Peru were examined.

Landmarks were determined by the intersection or termination of wing veins as follows:

1) junction of humeral and costal veins; 2) subcostal break along costal vein; 3) apex of vein R_1_; 4) apex of vein R_2+3_; 5) apex of vein R_4+5_; 6) apex of vein M; 7) apex of vein CuA_1 _on posterior margin; 8) apex of vein CuA_2_ on posterior margin; 9) basal bifurcation of R_2+3 _and R_4+5_; 10) junction of R_4+5_ and cross vein r-m; 11) basal angle of cell bm; 12) junction of M and cross-vein dm-bm; 13) junction of M and cross vein r-m; 14) junction of M and cross-vein dm-cu; 15) junction of CuA_1_ and Cu_2_; 16) junction of CuA_1_ and cross vein bm-cu; 17) junction of CuA_1_ and dm-cu; 18) junction of A and Cu_2_ (= apex of cell bcu) (Figure 4).

### Data analyses

Linear measurements and the landmark coordinates were acquired from digitized images of wing, aculeus and mesonotum using the TPS DIG software package (Rohlf 2010a, 2010b). Canonical Variate Analyses (CVA) were executed to explore the morphological similarities among the 40 populations of the *Af* complex, and to test the reliability of the predictive model of morphotypes as well. The model based on linear morphometry was constructed by the forward stepwise analysis method, which reviews all variables and evaluates which ones will contribute further to the discrimination between groups. From linear morphometric data, a dendrogram of the relationships among samples was constructed, based on Mahalanobis distances computed from the CVA by the unweighted pair group average method (UPGMA), using Statistica (Statsoft 2006). Statistical validation of morphotypes and lineages was made through multivariate analysis of variance (MANOVA) of the scores from the CVA’s, and their pairwise comparisons by Hotelling’s test with Bonferroni correction using R software (R Core Team 2014). Additional tests on the feasibility of the prediction model were performed through the classification function analysis of individuals grouped by morphotypes and lineages.

The wing shape information was extracted by the generalized Procrustes superimposition analysis, which is used to remove non-shape variation by scaling all specimens to unit size, translating to a common location and rotating them to their corresponding landmarks lined up as closely as possible (Goodall 1991). To test the accuracy of morphotypes and lineages established *“a priori”*, we conducted CVA’s and the classification of individuals by group using SPSS v.13 program. To prove their statistical significance, we also executed MANOVA tests and their pairwise comparisons (R Core Team 2014). To evaluate the effect of wing size, a multivariate regression of the wing shape (dependent variable) *vs.* log-centroid size (independent variable) with permutation tests (10,000 iterations) were performed. Differences of the wing shape were visualized using wireframe comparisons along the first two canonical variates. Procrustes superimposition analysis, wing size analysis, and drawing of wireframes were executed with MORPHOJ (Klingenberg 2011).

## Results

### Morphotypes

**Figure 5.**
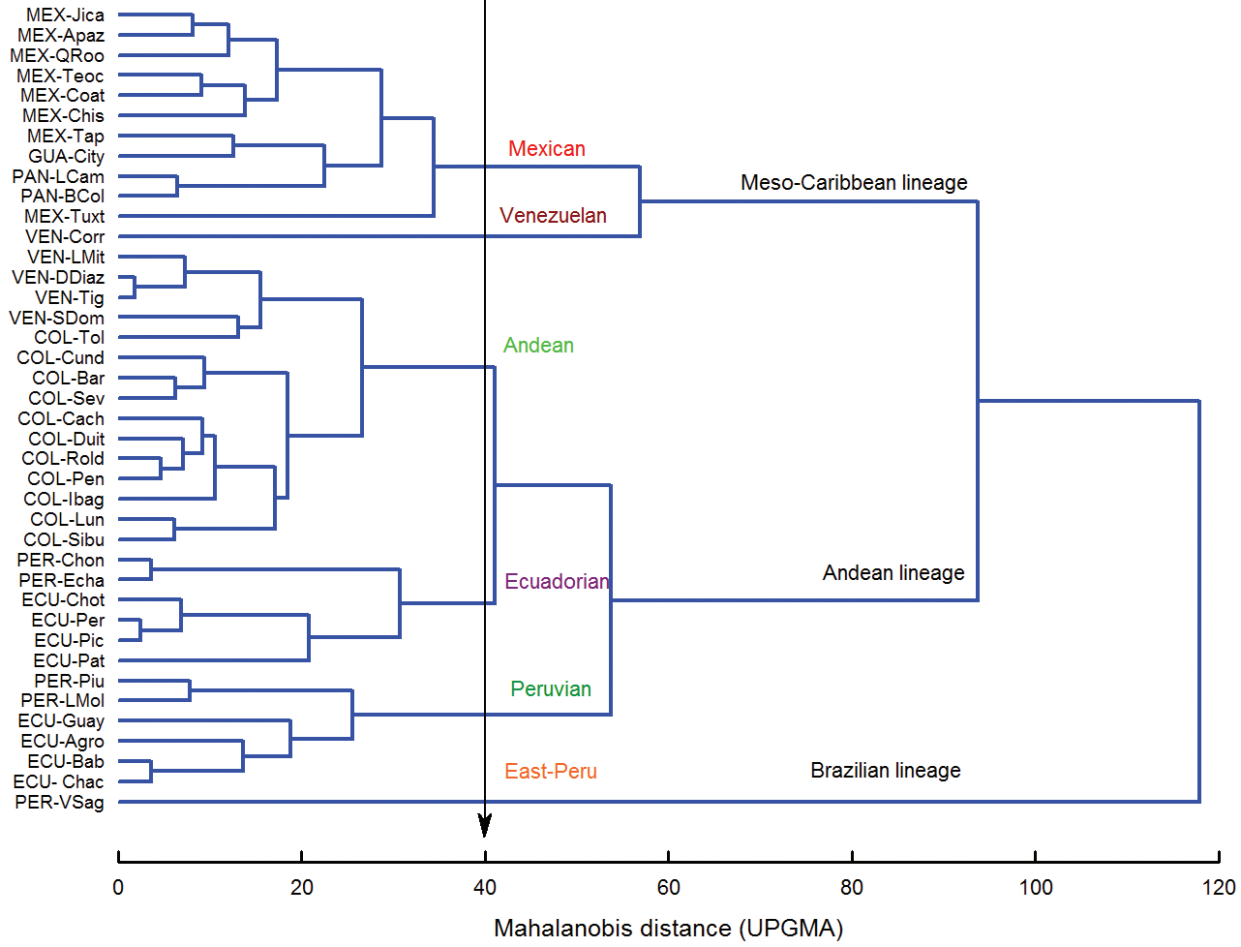
Phenogram showing the linear morphometric relationships among 40 population samples of the *Af* complex, based on Mahalanobis distances computed from the CVA. Clustering method UPGMA.

The exploratory canonical variate analysis (CVA) of linear morphometrics, applied to 40 populations along the Mesoamerican and Pacific Neotropical dominions showed significant differences among them (*F* = 9.40; Wilk’s lambda < 0.0001; DF = 39/547; *p* < 0.0001). The tree of similarities computed from the Squared Mahalanobis distance matrix, supported the presence of six well-differentiated morphotype clusters: the Mexican, Venezuelan, Andean, and Peruvian (previously established by Hernández-Ortiz et al. 2004, 2012), a new cluster designated as Ecuadorian morphotype, and a single population from East-Peru (Figure 5).

**Figure 6.**
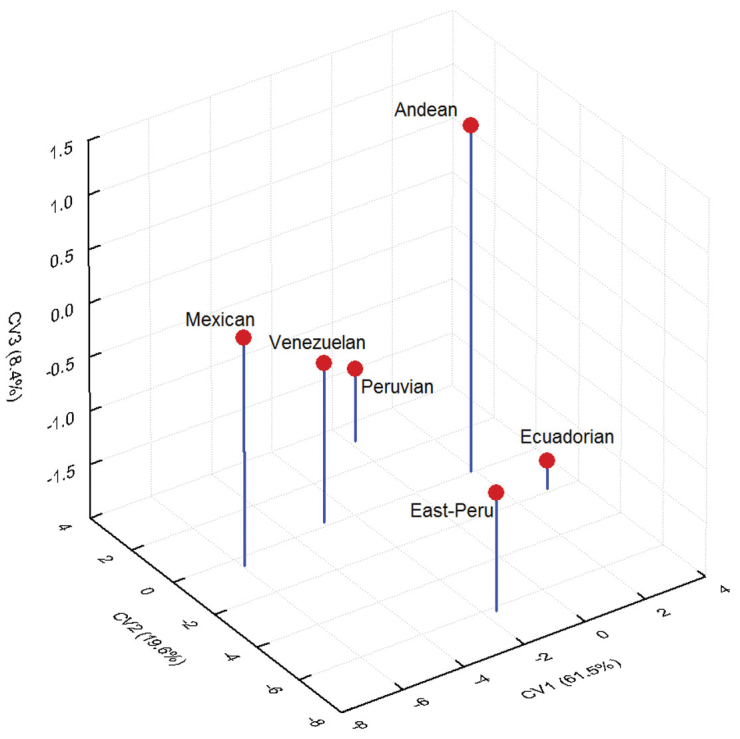
Scatter plot of means of centroids morphotypes from first three Canonical Variates resulted from the CVA applied to linear morphometric model. Percentage in parenthesis indicates the contribution to the differentiation of the groups.

The predictive model of linear morphometrics showed that centroid means for the Andean, Peruvian and Ecuadorian morphotypes were mainly differentiated by the CV-1 scores, which contributed with 61.5% of the differentiation. The CV-2 accounted for 19.6%, distinguishing the East-Peru sample. The CV-3 scores accounted for only 8.4% of the variation among groups (Table 2, Figure 6). Variables with greatest statistical significance were represented by characters of the aculeus (A1, A2, A7, A9), mesonotum (M1, M3), wings (W3, W4, W5), and ratios between two variables (X2, X3, X5) (Table 3).

**Table 2.** Chi-Square tests with successive roots removed. Results of five significant variates produced by two morphometric models.

Model	Function	Eigenvalue	Canonical R	% Variance	% Cumulative
Linear morphometrics	1	10.451	0.955	61.5	61.5
	2	3.333	0.877	19.6	81.1
	3	1.442	0.768	8.4	89.5
	4	1.156	0.732	6.8	96.3
	5	0.624	0.620	3.7	100
Geometric morphometrics	1	2.996	0.866	43.5	43.5
	2	2.171	0.827	31.5	75.0
	3	1.295	0.751	18.8	93.8
	4	0.309	0.486	4.5	98.3
	5	0.120	0.328	1.7	100.0

**Table 3.** Means and Standard Deviations for all measurements of the morphotypes encountered. Linear measures are in mm, except qualitative traits (W5, W6, A7), and ratios (A9, A10, A11, W7, X1, X2, X3, X4, X5, X6). See methods for explanations.

	Mexican	Venezuelan	Andean	Peruvian	Ecuadorian	East-Peru
A1	1.773 ± 0.10	1.945 ± 0.05	1.801 ± 0.10	1.68 ± 0.07	1.905 ± 0.12	1.727 ± 0.06
A2	0.123 ± 0.01	0.131 ± 0.01	0.123 ± 0.01	0.120 ± 0.01	0.136 ± 0.01	0.116 ± 0.01
A3	0.087 ± 0.01	0.093 ± 0.01	0.080 ± 0.01	0.079 ± 0.01	0.083 ± 0.01	0.077 ± 0.01
A4	0.117 ± 0.01	0.142 ± 0.01	0.120 ± 0.01	0.114 ± 0.01	0.128 ± 0.01	0.122 ± 0.01
A5	0.161 ± 0.01	0.178 ± 0.01	0.126 ± 0.01	0.132 ± 0.01	0.132 ± 0.01	0.143 ± 0.01
A6	0.284 ± 0.02	0.328 ± 0.02	0.253 ± 0.02	0.252 ± 0.01	0.268 ± 0.02	0.271 ± 0.02
A7	11.83 ± 1.52	14.13 ± 0.77	10.97 ± 1.16	13.11 ± 1.12	10.80 ± 0.97	9.65 ± 0.63
A8	0.277 ± 0.02	0.32 ± 0.01	0.250 ± 0.08	0.246 ± 0.01	0.260 ± 0.02	0.265 ± 0.02
A9	0.730 ± 0.10	0.803 ± 0.06	0.954 ± 0.14	0.866 ± 0.10	0.974 ± 0.14	0.856 ± 0.11
A10	0.157 ± 0.01	0.165 ± 0.01	0.139 ± 0.04	0.146 ± 0.01	0.137 ± 0.01	0.153 ± 0.01
A11	0.420 ± 0.03	0.445 ± 0.02	0.485 ± 0.04	0.463 ± 0.03	0.491 ± 0.04	0.460 ± 0.03
W1	6.287 ± 0.51	7.033 ± 0.26	6.653 ± 0.50	6.383 ± 0.31	7.089 ± 0.35	7.521 ± 0.41
W2	2.681 ± 0.24	2.903 ± 0.12	2.837 ± 0.23	2.785 ± 0.17	3.002 ± 0.15	3.126 ± 0.20
W3	0.441 ± 0.04	0.411 ± 0.03	0.314 ± 0.04	0.366 ± 0.03	0.300 ± 0.03	0.454 ± 0.03
W4	1.401 ± 0.13	1.459 ± 0.10	1.317 ± 0.17	1.429 ± 0.18	1.749 ± 0.11	1.936 ± 0.13
W5	1.16 ± 0.37	1.93 ± 0.26	1.98 ± 0.12	2.00 ± 0.00	2.00 ± 0.00	1.59 ± 0.51
W6	1.00 ± 00	1.00 ± 0.00	1.70 ± 0.46	1.77 ± 0.42	1.62 ± 0.49	1.00 ± 0.00
W7	0.426 ± 0.01	0.413 ± 0.01	0.427 ± 0.02	0.436 ± 0.01	0.424 ± 0.01	0.415 ± 0.01
M1	2.884 ± 0.24	3.159 ± 0.12	2.879 ± 0.26	3.061 ± 0.17	3.083 ± 0.20	3.005 ± 0.22
M2	1.900 ± 0.16	2.103 ± 0.09	1.856 ± 0.19	1.987 ± 0.11	2.036 ± 0.13	1.984 ± 0.14
M3	1.815 ± 0.15	2.007 ± 0.08	1.792 ± 0.19	1.925 ± 0.12	1.992 ± 0.12	1.910 ± 0.15
X1	0.617 ± 0.04	0.616 ± 0.02	0.628 ± 0.04	0.550 ± 0.03	0.621 ± 0.06	0.576 ± 0.04
X2	0.283 ± 0.02	0.277 ± 0.01	0.271 ± 0.01	0.263 ± 0.01	0.269 ± 0.02	0.231 ± 0.02
X3	0.459 ± 0.02	0.449 ± 0.01	0.433 ± 0.02	0.479 ± 0.02	0.434 ± 0.02	0.399 ± 0.02
X4	1.520 ± 0.09	1.503 ± 0.04	1.555 ± 0.06	1.541 ± 0.06	1.518 ± 0.08	1.515 ± 0.05
X5	0.540 ± 0.04	0.523 ± 0.04	0.635 ± 0.05	0.598 ± 0.04	0.630 ± 0.07	0.537 ± 0.04
X6	0.303 ± 0.02	0.299 ± 0.01	0.279 ± 0.01	0.311 ± 0.01	0.287 ± 0.01	0.264 ± 0.01
**Valid N**	**123**	**15**	**258**	**101**	**98**	**17**

MANOVA tests showed significant overall differences among morphotypes based on linear morphometry (*F* = 366.73; Wilk’s lambda = 0.0024; DF = 25/2237; *p* < 0.0001), and all Hotelling’s pairwise comparisons (*post hoc*) also led to significant differences (*p* < 0.0001). Furthermore, reliability of morphotypes based on the wing shape also proved to be statistically significant (*F* = 159.93; Wilk’s lambda = 0.0235; DF = 25/2289; *p* < 0.0001), as well as all Hotelling’s pairwise comparisons between each other (*p* < 0.0001).

Morphological similarities through the Squared Mahalanobis Distance matrix (SMD) were assessed by pairwise comparisons among morphotypes. For example, closer distances were noted between morphs such as Ecuadorian *vs.* Andean (SMD = 15.2), and Peruvian *vs.* Andean (SMD = 19.7); or moderate distances between Peruvian *vs.* Ecuadorian (SMD = 37.9), and Mexican *vs.* Venezuelan (SMD = 37.7) (Table 4). The overall rate of reliability to identify individuals within expected morphotypes was very high (96.1%). The correct classification of the specimens according to cross-validation of the CVA was 93.5 and 100% for Mexican and Venezuelan morphotypes, respectively; a range from 96.1–97% for the Andean, Peruvian and Ecuadorian morphotypes; while 100% of individuals from the East-Peru location (Per- Valle Sagrado) were correctly classified (Table 5).

**Table 4.** Squared Mahalanobis Distances from linear morphometric data produced by pairwise comparisons among morphotypes from Mesoamerica and Pacific Neotropical dominions.

	Mexican	Venezuelan	Andean	Peruvian	Ecuadorian	East-Peru
**Mexican**	0	37.7	62.8	54.6	87.0	84.3
**Venezuelan**		0	50.8	48.1	67.0	100.7
**Andean**			0	19.7	15.2	81.2
**Peruvian**				0	37.9	108.0
**Ecuadorian**					0	80.1
**East-Peru**						0

**Table 5.** Classification matrix of individuals by morphotypes according to tested models: Above line: Linear morphometrics. Below line: Geometric morphometrics. Rows: observed classifications; Columns: Predicted classifications.

	% Correct	Mexican	Venezuelan	Andean	Peruvian	Ecuadorian	East-Peru	N
Mexican	93.5	**115**	7	1	0	0	0	123
Venezuelan	100.0	0	**15**	0	0	0	0	15
Andean	96.1	0	0	**248**	1	9	0	258
Peruvian	97.0	0	0	3	**98**	0	0	101
Ecuadorian	96.9	0	0	3	0	**95**	0	98
East-Peru	100.0	0	0	0	0	0	**17**	17
**Linear model**	**96.1**	**115**	**22**	**255**	**99**	**104**	**17**	**612**
Mexican	87.8	**108**	9	0	4	0	2	123
Venezuelan	100.0	0	**15**	0	0	0	0	15
Andean	87.4	6	5	**229**	4	18	0	262
Peruvian	95.2	0	0	1	**100**	4	0	105
Ecuadorian	88.5	0	0	11	1	**92**	0	104
East-Peru	100.0	0	0	0	0	0	**17**	17
**Geometric model**	**89.6**	**114**	**29**	**241**	**109**	**114**	**19**	**626**

**Figure 7.**
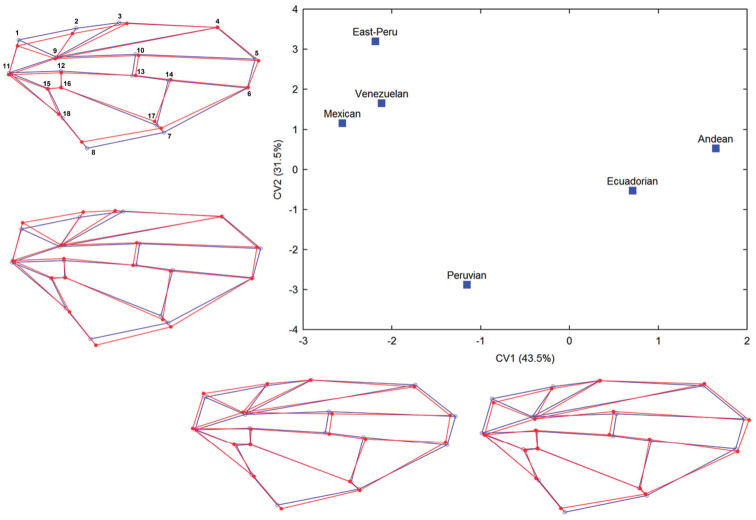
Average scores for the first two canonical variates (CV1 and CV2) derived from CVA for the total variation of wing shape between morphotypes of the *Af* complex. Wireframes showing the shape changes (red lines) from the consensus configuration of landmarks (blue lines) to each extreme negative and positive of CV scores.

Moreover, the predictive model based on the CVA of the wing shape showed that 43.5% of the variability can be explained by the first canonical variable (CV-1), which recognizes the closely linked Andean and Ecuadorian morphotypes, and in turn, is clearly divergent from others. The second canonical variable (CV-2) described 31.5% of differences, recognizing the Mexican and Venezuelan morphotypes near each other, but differing from the Peruvian morphotype. The third canonical variable (CV-3) accounted for only 18.8% of the variability among groups. These wing shape variations are represented by the wireframes of morphotypes encountered, showing the change of the shape expected along the first two canonical variables (Table 2, Figure 7).

**Figure 8.**
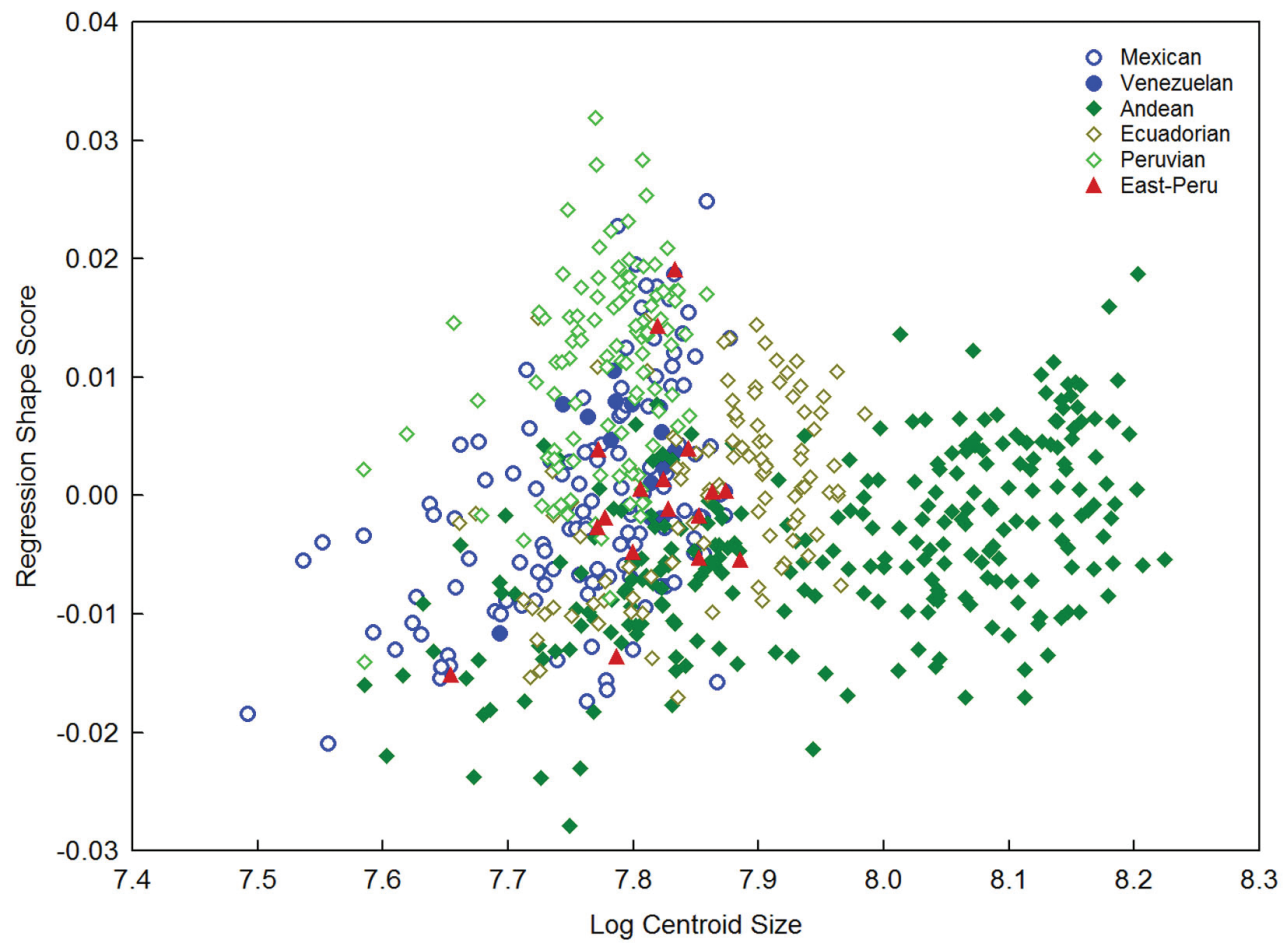
Analysis of allometry among morphotypes in the *Anastrepha fraterculus* complex. Multivariate regression performed from Procrustes coordinates against log-Centroid size values for the wings.

The allometric variation of the wing shape assessed by multiple regression of log-centroid size *vs.* shape scores, revealed significant differences (*p* < 0.0001), proving that wing size predicted for only 2.26% of the total shape variation. However though this test proved to be significant it is considered relatively minor given the low percentage shown (Figure 8). *A priori* allocation of individuals into each of the morphotypes resulted in an overall rate of 89.6% with some differences respect to linear model; the Andean and Ecuadorian morphotypes exhibited identification rates of 87.4% and 88.5%, respectively; the Mexican 87.8%, the Peruvian 95.2%, while in the Venezuelan and the East-Peru samples 100% of the specimens were correctly classified (Table 5).

### Phenotypic relationships

In accordance with the results from previous cluster analysis of the 40 populations examined, morphotypes were linked at higher distance forming three different phenotypical groups herein called the Meso-Caribbean, Andean and Brazilian phenotypic lineages. Multivariate regression analysis (MANOVA) applied to scores obtained from the CVA, proved that accuracy of lineages was highly significant. The linear model showed highly significant differences between lineages (*F* = 1150.8; Wilk’s lambda = 0.0437; DF = 4/1216; *p* < 0.0001), and among all pairwise comparisons (Hotelling’s test *p* < 0.0001). The predictive model, using the geometric method, also demonstrated highly significant differentiation between lineages (*F* = 433.3; Wilk’s lambda = 0.1746; DF = 4/1244; *p* < 0.0001) and all paired comparisons among them as well (Hotteling’s test, *p* < 0.0001). Mahalanobis distances exhibited remarkable divergence when contrasting morphotypes from distinct lineages; for instance, pairwise comparisons between East-Peru (Brazilian lineage) with all other morphotypes (SMD = 80.1–108), or distances among samples from the Andean lineage *vs.* the Meso-Caribbean lineage (SMD = 48.1–87.0) (Table 4).

**Figures 9–11.**
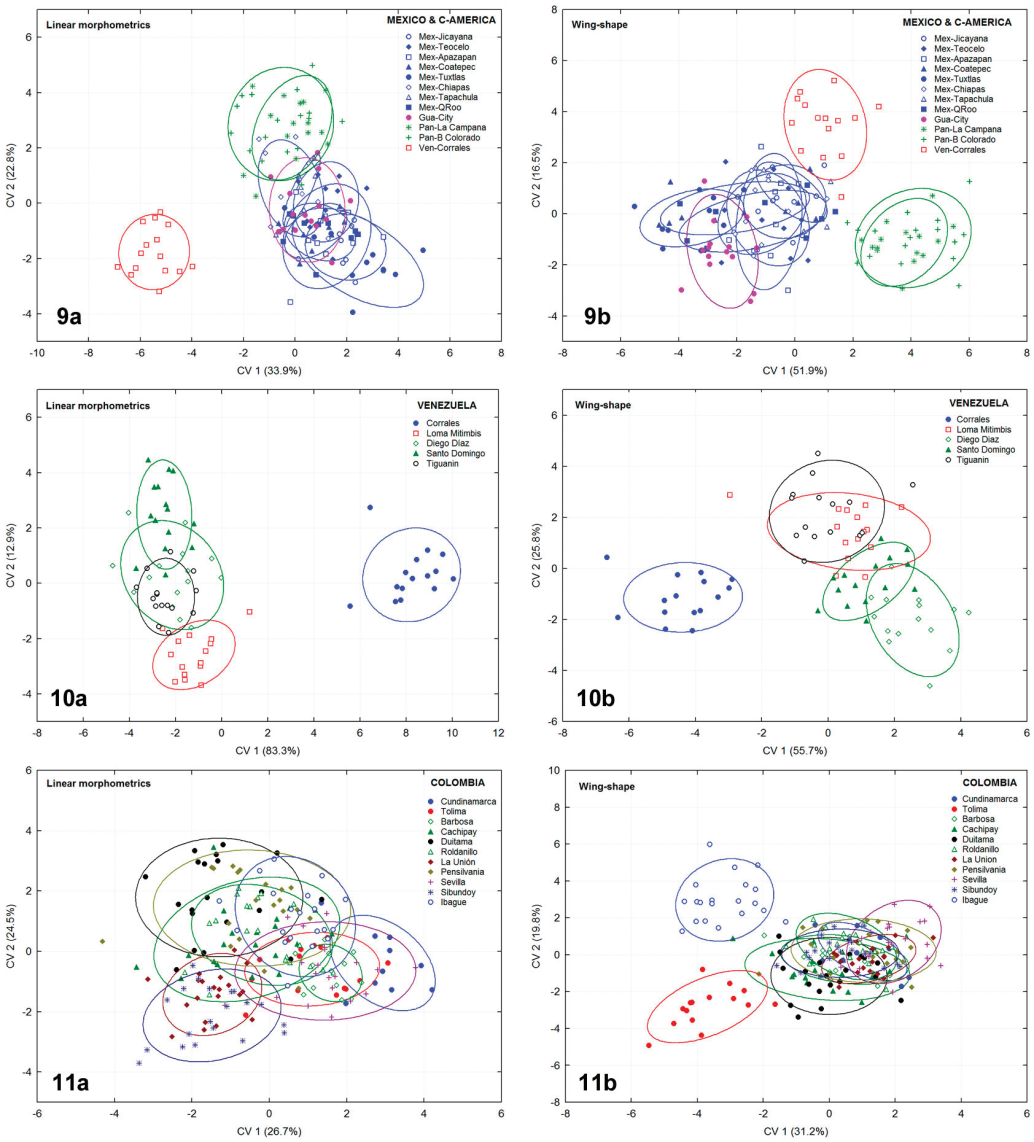
Scatter plots of individuals tested by CVA grouping samples by distributional areas: **9a–b** Mesoamerican-Caribbean lineage represented by 12 populations from Mexico, Guatemala, and Panama, including the single Venezuela lowland for comparisons **10a–b** Five populations from Venezuela **11a–b** Eleven populations from Colombia **a** linear morphometrics **b** geometric morphometrics of wing-shape. Confidence ellipses 95%.

*Mesoamerican-Caribbean lineage* (shortly named *Meso-Caribbean*). It clustered all samples from Mexico, Central America, and the Caribbean coast of Venezuela. This lineage consisted of the two vicariant Mexican and Venezuelan morphotypes (*sensu* Hernández-Ortiz et al. 2012). The former occurs in the territories of Mexico and Central America, and a single population from the Caribbean coast of Venezuela (Zulia state) distinguished the latter. The linear model showed close similarities among all samples from Mexico, Guatemala and Panama, and a clear segregation of the Caribbean population (Ven-Corrales). The wing shape model also exhibited similar results, nevertheless keeping separated the Panamanian populations (Figure 9a–b).

**Figures 12–13.**
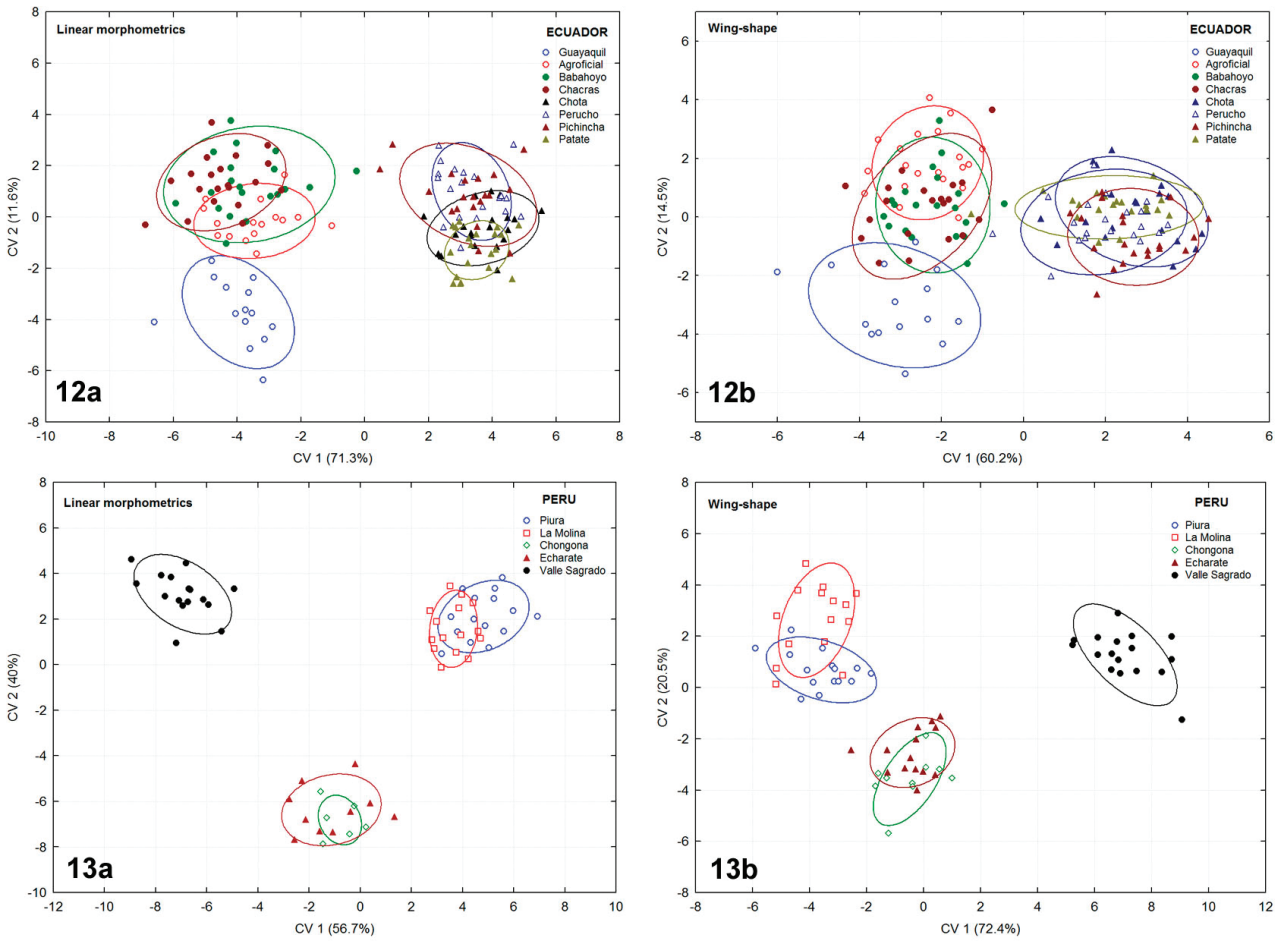
Scatter plots of individuals tested by CVA grouping samples by distributional areas: **12a–b** Eight populations from Ecuador **13a–b** Five populations from Peru. **a** linear morphometrics **b** geometric morphometrics of wing-shape. Confidence ellipses 95%.

**Figures 14–25.**
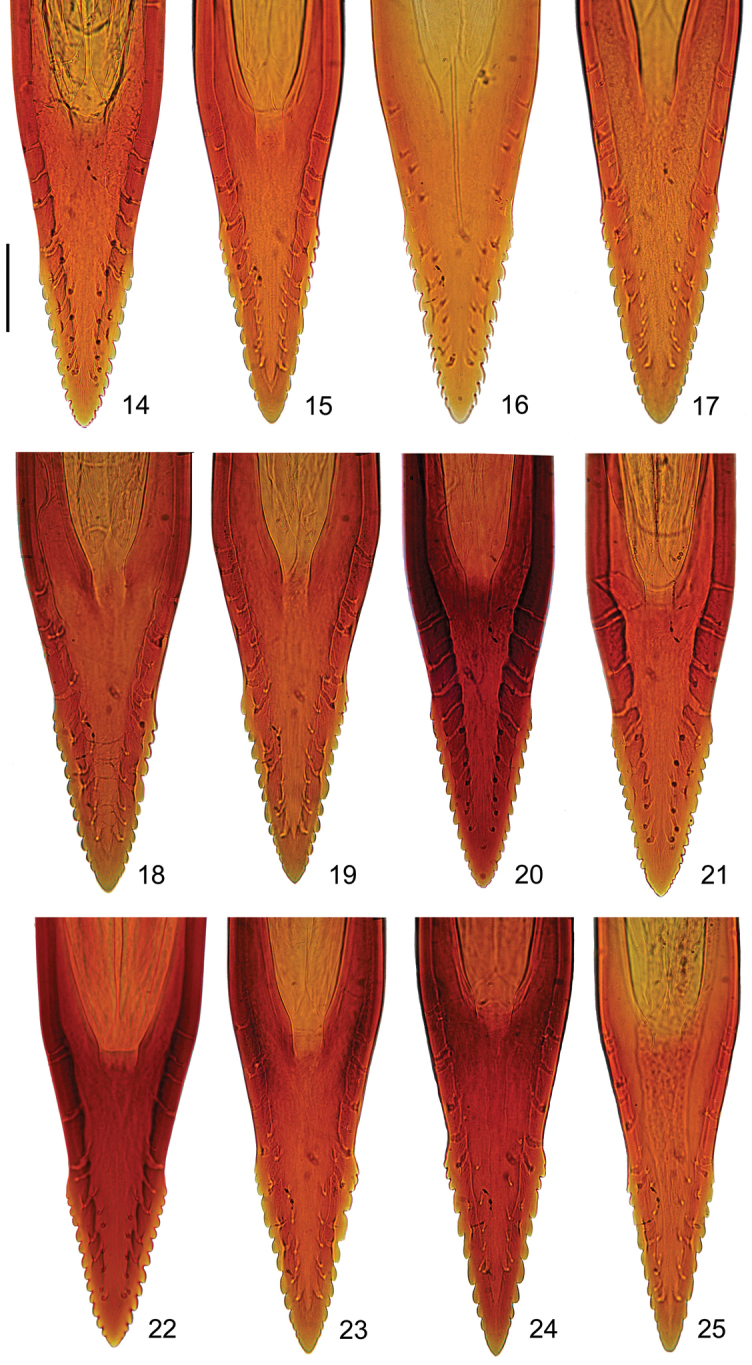
Typical shape of the acuelus tip in morphotypes from the Meso-American and Pacific dominions. Mexican morphotype: **14** Mexico-Apazapan **15** Guatemala-City **16** Panama-La Campana. Venezuelan morphotype: **17** Venezuela-Corrales. Andean morphotype: **18** Venezuela-Loma Mitimbis **19** Colombia-Barbosa. Peruvian morphotype: **20** Ecuador-Agroficial **21** Peru-La Molina. Ecuadorian morphotype: **22** Ecuador-Chota **23** Peru-Echarate. Brazilian lineage: **24** Peru-Valle Sagrado **25** Argentina-Tucuman. Scale bar 0.05 mm.

**Figures 26–37.**
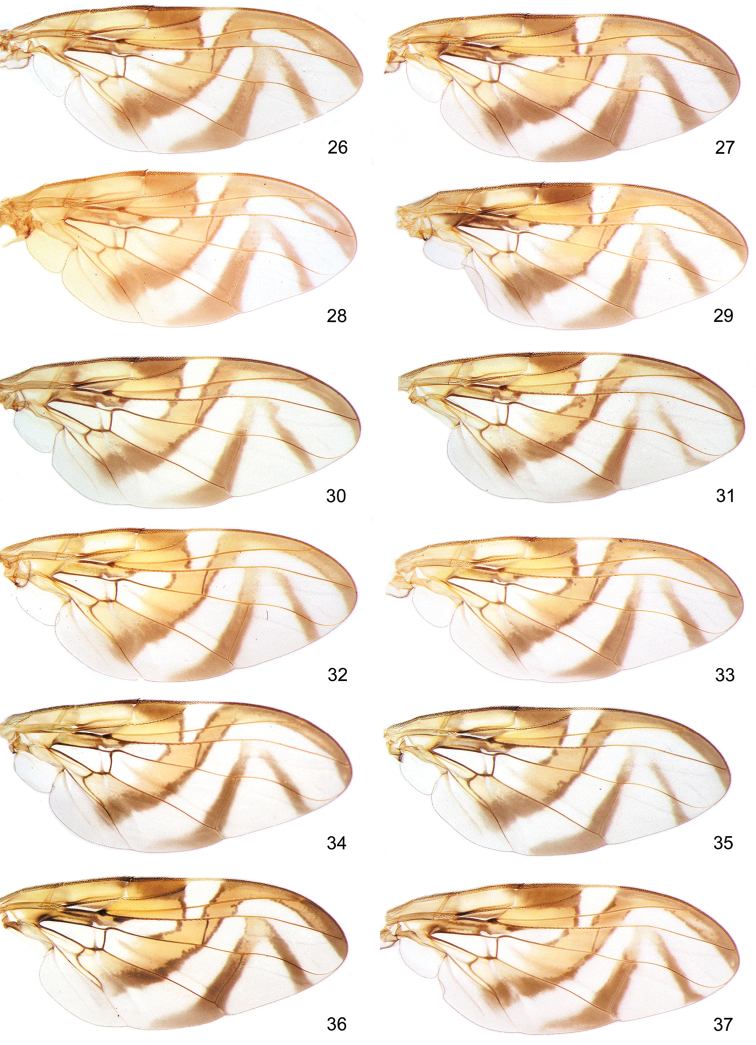
Typical wing patterns in morphotypes from the Mesoamerican and Pacific dominions. Mexican morphotype: **26** Mexico-Apazapan **27** Guatemala-City **28** Panama-La Campana. Venezuelan morphotype: **29** Venezuela-Corrales. Andean morphotype: **30** Venezuela-Loma Mitimbis **31** Colombia-Barbosa. Peruvian morphotype: **32** Ecuador-Agroficial **33** Peru-La Molina. Ecuadorian morphotype: **34** Ecuador-Chota **35** Peru-Echarate. Brazilian lineage: **36** Peru-Valle Sagrado, **37** Argentina-Tucuman.

This lineage exhibited distinctive morphological features such as the aculeus length (A1 = 1.77–1.95 mm); wider aculeus tip at beginning of serrated section (A3 = 0.087–0.093 mm); longer serrated section (A5 = 0.161–0.178 mm); ratio of non-serrated section/aculeus tip (A11 = 0.420–0.445); and lowest ratio of width/length of serrated section (X5 = 0.523–0.540), like specimens of the Brazilian lineage. Remarkable qualitative features in the wing pattern were also recorded: the typical Costal, S- and V- bands are broad and heavily colored; the upper connection between arms of V- band (W6) in nearly 100% of specimens examined; and wider apical section of S- band (W3 = 0.411–0.441 mm). In the Mexican morphotype, aculeus tip constriction at beginning of serrated section is almost unnoticeable, and connection between S- and V- bands is always present; whereas in the Venezuelan morphotype S- and V- band connection is typically absent in most specimens, and the aculeus tip wider with numerous marginal teeth (A7 = 14.1 teeth per side) (Figures 14–17, 26–29).

*Andean lineage*. It comprises three clusters of samples: a) the Andean morphotype grouped all 15 populations coming from high mountains of Venezuela and Colombia; b) the Peruvian morphotype clustered six lowland populations along the Pacific coast of Ecuador and Peru; and c) the Ecuadorian morphotype, here recognized by the first time, including six highland populations from Ecuador and Peru. We highlight some variables, which may distinguish the morphs of this lineage from others: the apical section of S- band extremely narrow (W3 = 0.300–0.366 mm); S- and V- band connection (W5) missing in near 97% of the specimens examined; V- band arms upper connection (W6) absent in nearly one half of the specimens; and higher ratio between width/length of serrated section (X5 = 0.598–0.635). The Peruvian morph exhibited higher average teeth on the aculeus tip (A7 = 13.1 teeth per side) when compared to Ecuadorian and Andean morphotypes (A7 = 10.8, 10.9 teeth per side, respectively). The Andean morph showed a strong narrowing of apical section of S- band, in addition to distal arm of V- band diffuse and reduced (Figures 18–23, 30–35).

*Brazilian lineage*. It was recognized by a single population from the high mountains of the East-Andean region in Peru, which showed a clear differentiation from all other samples studied, and a preliminary analysis placed it closely related to the Brazilian morphs (*sensu* Hernández-Ortiz et al. 2012). We have not fully characterized this lineage, since other Brazilian morphotypes were not reviewed under this perspective. However, we made some comparative inferences from other lineages here described. The sample from East-Peru (Per-Valle Sagrado) exhibited particular morphological traits as follows: shorter aculeus length; aculeus tip narrow and longer in proportion to total length; lowest number of marginal teeth (A7 = 9.6 teeth per side), this last one probably a common feature in populations inhabiting southern Brazil and Argentina (see Hernández-Ortiz et al. 2012). Ratios of the aculeus/wing length (X2 = 0.231), and mesonotum/wing length (X3 = 0.399) showed the lowest values among all samples. This means that wing length is larger than aculeus and mesonotum respect to other morphotypes examined (Figures 24–25, 36–37).

### Distribution

The dendrogram of morphometric similarities also provided evidence that more than one morphotype could occur in some South American countries located in the Pacific dominion. Therefore, further discriminant analyses were performed separately.

*Venezuela*. Samples from five locations were considered for the analyses and the results from both linear and geometric morphometry were almost identical. The single population examined of the Caribbean coast (Ven-Corrales) belonged to the Venezuelan morphotype (Meso-Caribbean lineage), and it was distinguished from a second group comprising all four populations coming from the highlands, identified as the Andean morphotype (Andean lineage) (Figure 10a–b).

*Colombia*. Linear morphometric analysis grouped all 11 Colombian populations under the Andean morphotype. Nevertheless, the wing shape analysis revealed three partially differentiated groups: one cluster with individuals from 9 populations, a second sluster with individuals from the laboratory strain of the Vienna facilities (Col-Tolima), and the other one from Ibagué (Col-Ibagué) (Figure 11a–b).

*Ecuador*. The linear morphometrics and wing shape analyses applied to eight populations from Ecuador yielded identical results, forming two distinct morphological clusters inhabiting this country. The four lowland samples were closely related to each other within the Peruvian morphotype (*sensu* Hernández-Ortiz et al. 2012), while the four other samples from the highlands were classified under a new Ecuadorian morphotype, here described for the first time (Figure 12a-b).

*Peru*. Both morphometric techniques applied to five populations analyzed of this country revealed the presence of three different morphological clusters. The first one comprised two lowland samples classified into the Peruvian morphotype (Per-Piura, Per-La Molina). The second cluster was represented by two samples from the highlands (Per-Echarate, Per-Chongona) and belonged to the Ecuadorian morphotype. The third morphological entity, consisting of a single population from the East-region of the Andes (Per-Valle Sagrado), proved to be distinct from all other samples examined, tentatively related to the Brazilian-1 morphotype within the Brazilian lineage (Figure 13a–b).

Distributional patterns based in the current classification of the Neotropical biogeographic provinces (*sensu* Morrone 2014), showed that the Mexican morphotype occurs in areas from 30–1400 m altitude in the Veracruzan, the Pacific lowlands, and the Yucatan peninsula provinces (Mesoamerican dominion); also in the Chiapas highlands province (Mexican Transition Zone), and in Central America recorded from the Guatuso-Talamanca province (north of the Pacific dominion), and probably spread to the Puntarenas-Chiriquí province (Table 6). The Venezuelan morphotype was recorded from the Guajira province (40 m); however, it could reach out other nearby lowlands along the Caribbean coast into the Venezuelan province, as represented by lowland samples near Caracas examined by Steck (1991), and Steck and Sheppard (1993).

**Table 6.** Distribution of the morphotypes through biogeographical provinces of the Mesoamerican and Pacific dominions (*sensu* Morrone 2014).

Morphotype	Biogeographical Sub-region	Biogeographical Province	Country	Sample-Key
**Mexican**	Mesoamerica	Veracruzan	Mexico	MEX-Jica
			Mexico	MEX-Teoc
			Mexico	MEX-Apaz
			Mexico	MEX-Coat
			Mexico	MEX-Tuxt
		Pacific Lowlands	Mexico	MEX-Tap
		Yucatan Peninsula	Mexico	MEX-QRoo
	Mex Tran Zone	Chiapas Highlands	Mexico	MEX-Chis
			Guatemala	GUA-City
	Pacific	Guatuso-Talamanca	Panama	PAN-Lcam
			Panama	PAN-Bcol
**Venezuelan**	Pacific	Guajira	Venezuela	VEN-Corr
**Andean**	Pacific	Magdalena	Venezuela	VEN-Lmit
			Venezuela	VEN-DDiaz
			Venezuela	VEN-Sdom
			Venezuela	VEN-Tig
			Colombia	COL-Cund
			Colombia	COL-Tol
			Colombia	COL-Bar
			Colombia	COL-Cach
			Colombia	COL-Duit
			Colombia	COL-Pen
			Colombia	COL-Ibag
		Cauca (north)	Colombia	COL-Rold
			Colombia	COL-Lun
			Colombia	COL-Sev
			Colombia	COL-Sibu
**Ecuadorian**	Pacific	Cauca (south)	Ecuador	ECU-Chot
			Ecuador	ECU-Per
			Ecuador	ECU-Pich
			Ecuador	ECU-Pat
	South Brazilian	Yungas	Peru	PER-Chon
			Peru	PER-Echa
**Peruvian**	Pacific	Western Ecuador	Ecuador	ECU-Guay
			Ecuador	ECU-Agro
			Ecuador	ECU-Baba
			Ecuador	ECU- Chac
		Ecuadorian	Peru	PER-Piu
	S-Am Tran Zone	Desert	Peru	PER-LMol
Brazilian complex	S-Am Tran Zone	Puna	Peru	PER-VSag

The Andean morphotype only occurs in the Pacific dominion along the Magdalena province, occupying the highlands of Venezuela (from 1570–2500 m altitude) and Colombia (from 1350–2569 m); it was also found in several Colombian locations in the north of the Cauca province (Roldanillo, La Union, Sevilla, and Sibundoy). However, in the Colombian Pacific lowlands represented by the Chocó-Darién province, we did not record any sample of the *Af* complex so far.

The Peruvian morphotype was distributed throughout the Pacific Coastal lowlands from Ecuador (7–370 m) and Peru (35–300 m), into the Western-Ecuador and Ecuadorian provinces (Pacific dominion), and the Desert province of the South American Transition Zone. The Ecuadorian morphotype exhibited a distribution along the mountains of the south of Cauca province in the inter-Andean valleys from Ecuador (1550–2176 m), together with two other Peruvian highland samples (Per-Chongona, Per-Echarate) located at 941–1502 m, respectively, in the East-side of the Andes within the Yungas province (South Brazilian dominion). A single population sample was characterized as belonging to the Brazilian lineage, and it was collected in Cusco at the Inca region called Sacred Valley (2859 m), located in the East-side of the Andes into the Puna province of the South American Transition Zone.

## Discussion

Results showed that the nominal species *Anastrepha fraterculus* (Wiedemann) includes several cryptic species in concordance with previous morphometric findings (Hernández-Ortiz et al. 2004, 2012). Specifically, the *Af* complex consists of eight morphotypes throughout the Neotropics, and in turn, these are related to each other within at least three phenotypic lineages. Major similarities were seen between morphotypes belonging to the same lineage; for example, closer distances were observed between the Mexican and Venezuelan morphotypes (Meso-Caribbean lineage), or between the Andean and the Ecuadorian morphotypes (Andean lineage). The phenotypic proximity between the Ecuadorian and the Andean morphotypes, together with the fact that individuals were partially classified within each other, means that they could have a partial and incomplete isolation.

Linear and geometric morphometric analyses showed similar results, both demonstrating to be useful for diagnosis and recognition of morphotypes presumably representing the cryptic species of the *Af* complex. However, we should also mention that some differences were noted. For instance, differences between samples reared from laboratory colonies, originally stemmed from the same area in Colombia (Col-Tolima, Col-Ibagué) proved to be divergent in wing shape between each other. This is probably due to laboratory strains facing phenotypic selection under artificial conditions over many generations. Therefore, it is advisable to use wild samples for identification of natural morphs, especially if geometric morphometrics is applied. Wing shape analysis also differentiated two Panamanian samples (Pan-La Campana, Pan-B Colorado) from other populations belonging to the Meso-Caribbean lineage, even though they belonged to field collections. This highlights the need to further investigate other samples from that region to assess natural variation.

It could be argued however, that other factors may have influenced the ultimate morphological phenotype of the wing shape of flies. In particular, altitude has been found to have an impact on the wing shape of the potato moth (Hernández et al. 2010); wing shape differences between the fruit flies *Rhagoletis pomonella* and *Rhagoletis zephyria*, were hypothesized to have changed in relation to host associations (Yee et al. 2009). However, we consider this might not be the case within the *Af* complex for several reasons. Firstly, the nominal species *Anastrepha fraterculus* is highly polyphagous, and host usage, albeit not the same, is highly overlapping among morphotypes (Norrbom 2004, Hernández-Ortiz and Morales-Valles 2004, Zucchi 2007). For example, *Psidium guajava* L. is a host widespread along its geographic distribution and altitudinal range in Mexico, Central America, the Andean countries, Brazil and Argentina. Secondly, the morphometric analysis performed on *Anastrepha fraterculus* in Mexico distinguished a single Mexican morphotype, even though eight populations from a wide altitudinal range, and belonging to three distinct host species were examined (Hernandez-Ortiz et al. 2004). Thirdly, in the Brazilian territory there are proofs of the occurrence of three morphotypes (Hernández-Ortiz et al. 2012), and evidence of karyotype differentiation and reproductive isolation supporting the existence of distinct species (Selivon et al. 2004, 2005a, 2005b); however all of them feed on guava, among other hosts.

Species boundaries are related with the extent and limits of gene flow, the selection intensities on ecologically or reproductively functional phenotypes across the species range, and their genetic architecture, all indispensable pieces of information for predicting the course of early lineage divergence and the origins of new species (Shaw 1998). In the biological species concept defined as “groups of interbreeding natural populations that are reproductively isolated from other such groups” (*sensu* Mayr 1969), it is not clear that in all sexually reproducing species, reproductive ties such as gene flow between demes provide the major cohesive force. The concept of interbreeding is a rather complex idea, because hybridization can be discussed in terms of reproductive modes but also in terms of speciation, that is, hybridization as *“prima facie”* evidence for incomplete speciation (Wiley 1981).

By contrast, in the evolutionary species concept defined as “a single lineage of ancestor – descendant populations, which maintains its identity from other such lineages and which has its own evolutionary tendencies and historical fate” (*sensu* Wiley 1978, modified from Simpson 1961), all terminal lineages are evolutionary species or descendant of higher taxa represented by their ancestral evolutionary species. Each branch therefore is the result of a speciation event; however, the concept does not preclude a particular ancestral species from surviving a speciation event. In this regard, De Queiroz (1998) noted that the term lineage is used for a single line of direct ancestry and descent, while a clade is a unit consisting of an ancestral species and its descendants, and are monophyletic in terms of their component species, however, lineages can be paraphyletic or even polyphyletic in terms of their lower level components.

In the broad sense, the monophyly of the *fraterculus* species group has been accepted based on morphology (Norrbom et al. 1999, Norrbom et al. 2012). In several papers it is assumed that reproductive isolation between cryptic species of the *Af* complex has recently evolved, leaving implicit the idea that those morphospecies had direct relationships, and at some time of its evolutionary history there was an interpopulation divergence among them (Cáceres et al. 2009, Segura et al. 2011, Rull et al. 2013, Devescovi et al. 2014). This is probably true among morphospecies related within each phenotypic lineage that could have a common origin and most likely a more recent evolution. However, from a theoretical perspective, the monophyly of the *Af* complex has never been tested so far. Conversely, other studies from various methodological sources stated that this species complex is not monophyletic. This assumption is supported by extreme allele differences found between highland and lowland Venezuelan samples (presumably from distinct lineages), being the largest genetic divergence found among samples studied by Steck (1991). The phylogenetic relationships inferred from mtDNA sequences of COI supported the presence of multiple gene pools and the non-monophyly among samples of the nominal species *Anastrepha fraterculus* (Smith-Caldas et al. 2001). In the same way, a phylogenetic relationship analysis based on the nuclear gene period of *Anastrepha* (Barr et al. 2005) with samples from Venezuela (Mérida and Caracas), Mexico, and Brazil (Sao Paulo) found them to be related in different clades.

The occurrence of strong sexual incompatibility between distinct phenotypic lineages also supports the non-monophyly hypothesis. For instance, high levels of pre- and post-zygotic isolation, karyotypic and polytene chromosome differences, and qualitative and quantitative differences in male pheromones were found in two laboratory strains from Argentina and Peru (Cáceres et al. 2009, Segura et al. 2011) which belong to the Brazilian and Andean lineages, respectively. In addition, pre-zygotic reproductive isolation resulted in strong assortative mating to gene flow among the Mexican morphotype and other populations classified in the Brazilian-1 and Peruvian morphotypes (Rull et al. 2013), all of them belonging to three distinct phenotypic lineages described herein. Moreover, there is strong pre-zygotic isolation through temporal partitioning of mating activity of a Colombian population (Andean morphotype) compared with four other morphotypes spanning from Mexico to Argentina (Devescovi et al. 2014).

In fact, the current study reveals that the *Af* complex is integrated by eight morphotypes, which are related into three phenotypic lineages that are virtually endemic, as they are restricted to certain regions, and there is no evidence of contact zones among them so far. The Meso-Caribbean lineage is restricted to the Mesoamerican dominion, to part of the Mexican Transition Zone, and also to the northern of Pacific dominion in Central America and the Caribbean coast of Venezuela. The Andean lineage essentially occupies most of provinces in the Pacific dominion and some parts of the South American Transition Zone; while the Brazilian lineage would be distributed along the Parana dominion in the eastern part of Brazil, and the Chacoan dominion in southern Brazil and northern Argentina.

In this regard, there are also historical processes associated to each biogeographical dominion that cannot be neglected, since they explain the own history of the biota they inhabit. According to Hoorn et al. (2010) plate subduction along the Pacific margin caused uplift in the Central Andes (Peruvian and Bolivian Andes) during the Paleogene (65 to 34 Ma). The posterior plate breakup in the Pacific, and subsequent collision with the South American and Caribbean plates, resulted in intensified mountain building in the Northern Andes (Venezuelan, Colombian and Ecuadorian Andes) by the late Oligocene to early Miocene (~ 23 Ma); while plate reorganization ultimately resulted in closing of the Panama Isthmus during the Pliocene (at ~ 3.5 Ma). These data sustain that the origin of the Northern and Central Andes, and their current connection with Mesoamerica, occurred in remarkable different times. This would mean prolonged periods of isolation between morphotypes inhabiting those geographical areas. In this sense, Drew (2004) stated that high levels of endemism in an area would indicate that speciation has occurred in relative isolation over a considerable time.

The relationship between morphological structure and genotype is complex and poorly understood for most characters, since we need to know if there is a relationship between the morphological characterizations and the real units of evolution (Shubin and Marshall 2000). This idea is particularly relevant when large numbers of sibling species occur. Therefore, from a practical point of view, it is necessary to understand the mechanisms of reproductive isolation between morphotypes, also as an essential precondition for applying control methods such as the sterile insect technique (SIT). However, from an evolutionary perspective, implications of the non-monophyly of the *Af* complex prevent making direct inferences about mechanisms of genetic or reproductive divergence among populations, since morphotypes belonging to distinct phenotypic lineages might have evolved independently in different clades.

## Conclusions

In this research, the presence of eight morphotypes is established within the *Anastrepha fraterculus* (Wiedemann) complex, including the first characterization of the Ecuadorian morphotype with samples coming from the mountains of Ecuador and Peru. The morphotypes clustered into three phenotypic lineages we called Meso-Caribbean, Andean, and Brazilian. Based upon their morphological divergence and the current distributional areas, we suggest that these lineages would not have a direct connection with each other and might have evolved separately in these biogeographical regions. In terms of distributional areas or countries, the Mesoamerican dominion was only occupied by the Mexican morphotype. In other countries from the Pacific dominion such as Colombia and Venezuela, two morphotypes were encountered, the Venezuelan inhabiting the Caribbean lowlands of Venezuela, and the Andean in the highlands of both countries. In the territories from Ecuador and Peru, the Peruvian morphotype was found in the lowlands, and the Ecuadorian morphotype in the highlands. Furthermore, in the Eastern side of the Andes in Peru, another morphotype was detected that appears closely related to the morphotypes of the Brazilian lineage.
